# Human papillomavirus testing with Pap triage for cervical cancer prevention in Canada: a cost-effectiveness analysis

**DOI:** 10.1186/1741-7015-7-69

**Published:** 2009-11-09

**Authors:** Shalini L Kulasingam, Raghu Rajan, Yvan St Pierre, C Victoria Atwood, Evan R Myers, Eduardo L Franco

**Affiliations:** 1 Duke University, Center for Clinical Health Policy Research, Durham, NC USA; 2 Dept of Obstetrics and Gynecology, Duke University Medical Center, Durham, NC, USA; 3 Divisions of Clinical Epidemiology and Medical Oncology, Departments of Oncology and Medicine, McGill University, Montreal, Quebec, Canada; 4 Division of Cancer Epidemiology, Departments of Oncology and Epidemiology & Biostatistics, McGill University, Montreal, Quebec, Canada; 5 Hepatitis C & STI Surveillance and Epidemiology Section Public Health Agency of Canada, 100 Eglantine Driveway, Bldg 6, 3428, AL 0603B, Ottawa, Ontario, K1A 0K9, Canada; 6 University of Minnesota, Twin Cities, Dept of Epidemiology,1300 S 2nd Street, Minneapolis, MN 55454, USA

## Abstract

**Background:**

Recently published results from a large randomized trial (Canadian Cervical Cancer Screening Trial study group) suggest that human papillomavirus testing followed by Pap smear-based triage for human papillomavirus positive women may be an effective way to screen women for cervical cancer. We determined the potential cost-effectiveness of including human papillomavirus tests for cervical cancer screening for Canada and three provinces: Alberta, Newfoundland and Ontario.

**Methods:**

We developed four Markov decision models using data from relevant Canadian and provincial studies and databases. The models were used to determine the number of false positive test results, cancers, lifetime costs and life-expectancy for 27 different screening strategies that varied by age to begin screening (18 or 25 years), screening interval (one, two, three, or five years) and whether the currently recommended strategy (screening every year from age 18 until 21 and then every three years afterwards with conventional Paps) was conducted prior to age 25. Strategies were compared using incremental cost-effectiveness ratios.

**Results:**

Screening strategies beginning at age 18 were associated with a substantial increase in the number of false-positive test results but only small differences in the number of cancers compared to the same strategy conducted beginning at age 25. Strategies of human papillomavirus testing first, followed by triage with Pap smears were associated with lower costs and greater increases in life-expectancy than the currently recommended screening strategy in Canada.

**Conclusion:**

A strategy of human papillomavirus testing beginning at age 25, with Pap triage for women with positive human papillomavirus results may be more effective at reducing cervical cancer at a lower cost than the current recommended strategy for screening in Canada.

## Background

In 2008, approximately 1,450 women were expected to be diagnosed with cervical cancer, and 420 women were expected to die from this disease in Canada [1]. The fact that Pap smear-based screening is offered to women in Canada beginning at age 18 until age 70 is attributed as the major reason for a reduction in cervical cancer mortality of almost 50% with respect to historical levels [2].

There are, however, continuing concerns with Pap smear-based screening, including the poor reproducibility of results, insufficient sensitivity and, as a result, the need for screening on a relatively frequent basis in order to achieve acceptable program sensitivity [3]. Human papillomavirus (HPV) is an established cause of cervical pre-cancer and cancer [4,5]. Studies have shown that HPV tests are significantly more sensitive, but less specific than Pap smear-based tests for the detection of cervical high-grade disease [6]. In Canada, HPV tests are currently recommended only as an adjunctive test for women with atypical cells of unknown significance (ASC-US) Pap smear results [7]. To determine whether HPV testing should be used in primary screening, a randomized trial was recently conducted (the Canadian Cervical Cancer Screening Trial [CCCaST]) [8,9] The aim of this trial was to determine the sensitivity and specificity of HPV testing either alone or in combination with Paps, compared to Pap tests only, for the detection of cervical intraepithelial neoplasia (CIN) grade 2 or 3 (CIN 2+). Published results from the trial show a significantly increased sensitivity of HPV testing for CIN 2+ (94.6, 95% CI: 84.2 - 100) compared to Pap tests (55.4%, 95% CI: 33.6 - 77.2), but a lower specificity (94.1%, 95% CI: 93.4 - 94.8 and 96.8%, 95% CI: 96.3 - 97.3) [8].

In this study, we used the estimates of test accuracy from CCCaST in conjunction with a previously published Markov model to determine the effectiveness and cost-effectiveness of 27 different screening strategies that included Pap tests only, HPV tests only or both Pap and HPV tests in combination [8,10]. Four cost-effectiveness models (one for Canada, and three provincial models for Alberta, Newfoundland and Ontario) were developed to determine whether a single, national strategy could be recommended or whether there was sufficient variation at the provincial level to consider different cervical cancer screening strategies by province.

## Methods

### Overview

#### Markov model of cervical carcinogenesis

We used a Markov model that simulates the natural history of cervical cancer in a theoretical cohort of women to estimate lifetime costs and life expectancy of different screening strategies. This Markov model has previously been described in detail [10-12]. Briefly, a population advances from one health state to another based on predefined probabilities. These health states are chosen to reflect the progression from healthy and disease-free through pre-cancer to cancer and death. Each year, women can either remain in the same state, progress to a more advanced disease state, or regress to a less severe disease state. Each year, women are also at risk of dying from causes other than cervical cancer or having a hysterectomy for a non-cancerous uterine condition.

#### Natural history

The model was calibrated to obtain an age-specific HPV prevalence curve based on large HPV epidemiology studies conducted in different Canadian settings; this was accomplished by varying the age-specific HPV incidence rates [13-16]. Progression and regression rates between the different states were from the original model by Myers et al. [10]. Benign hysterectomy rate was estimated from the Canadian Community Health Survey [17]. Death from non-cervical cancer causes was estimated from the Canadian Incidence and Mortality data set [18]. Cancer progression rates and the probability of symptoms were from Myers et al. [10]. Stage-specific survival probabilities were based on United States. Surveillance Epidemiology and End Results data from the National Cancer Institute [19]. The same underlying natural history was assumed for Canada as a whole and for each province.

#### Screening strategies

We compared 27 different screening strategies as summarized in Table 1. Strategies differed by the age at which to begin screening (either age 18 or age 25) and whether the most widely recommended strategy for cervical cancer screening in Canada was conducted prior to age 25 [20]. This baseline recommended strategy for Canada (screening every year from age 18 until 21 and then every three years afterwards until age 70 with conventional Pap tests) is hereinafter designated the 'Miller' strategy. The choice of age 25 was based on the fact that HPV testing prior to that age suffers from an unacceptably low specificity (to detect high grade cervical lesions) as a consequence of the high HPV prevalence following the first few years after the onset of sexual activity. This age may be an appropriate age to implement HPV DNA testing because HPV infections among such women are more likely to reflect underlying lesions [14]. In addition to single test (HPV or Pap) strategies, we examined a combined test strategy (HPV and Pap), in which women who were HPV positive or who had smears with findings of atypical squamous cells of undetermined significance (ASC-US) or more severe (≥ ASC-US) abnormalities were referred to colposcopy. We also examined two triage strategies: 1) HPV followed by Pap testing for HPV positive women and 2) Pap followed by HPV testing for women with ≥ ASC-US Pap test results. Adherence to screening recommendations by age was varied based on province-specific estimates provided by the 1998 Surveillance Report on Cervical Cancer Screening in Canada [2]. All women who received an initial screening test were also assumed to receive the triage test if a triage test was included as part of the strategy. Women with histologically confirmed CIN of grade 1 (CIN 1) were assumed to be followed with repeat Pap tests and to be treated if they had another abnormal test result (≥ ASC-US). Women with biopsy-confirmed CIN of grades 2 or 3 (CIN 2+) were assumed to receive loop electrosurgical excision procedure (LEEP). Adherence to follow-up and treatment was assumed to be 100%. Women with cancer were assumed to receive stage-specific treatment. Selected parameters used in the model are presented in Table 2.

**Table 1:** Twenty-seven screening test strategies that differ by age of first screening, type of screening test and frequency of screening.

*Test or test combination*	*Strategy*	*Frequency (years)*	Age when screening strategy begins		
			18 years	25 years	18-24 via Miller^1^, 25+ via strategy
Pap only	Pap^1^	1 (age 18 to 20) and 3 (age 21+)	X (Screening Strategy (SS) 1)		
	Pap	1	X (SS 2)		
	Pap	2	X (SS 3)		
HPV testing only^2^	HPV	3	X (SS 4)	X (SS 5)	X (SS 6)
	HPV	5	X (SS 7)	X (SS 9)	X (SS 9)
Co-testing^3^	Pap + HPV	2	X (SS 10)	X (SS 11)	X (SS 12)
	Pap + HPV	3	X (SS 13)	X (SS 14)	X (SS 15)
	Pap + HPV	5	X (SS 16)	X (SS 17)	X (SS 18)
Triage (Pap followed by HPV)^4^	Pap with HPV triage	1	X (SS 19)	X (SS 20)	X (SS 21)
Triage (HPV followed by Pap)^5^	HPV with Pap triage	3	X (SS 22)	X (SS 23)	X (SS 24)
	HPV with Pap triage	5	X (SS 25)	X (SS 26)	X (SS 27)

^1 ^This is the current recommendation for screening in Canada[20] Women are screened annually from age 18 to 20, and then every three years (for age 21+). Women who have an ≥ LSIL test result are referred for colposcopy and biopsy. Women with an ASC-US Pap result have a repeat Pap test and are referred to colposcopy and biopsy if their test result shows ≥ ASC-US. Women with a normal Pap test result return to routine screening. This strategy is referred to as the Miller strategy.

^2 ^Women who are HPV+ (using a 1 RLU cutpoint) are referred directly for colposcopy and biopsy. Women who are HPV- return to routine screening.

^3 ^Women who are HPV+ or who have an ≥ ASC-US Pap result are referred directly for colposcopy and biopsy. Women who are HPV- and have a normal Pap test result return to routine screening.

^4 ^Women with an ≥ ASC-US Pap result are assumed to have an HPV test. Women who are HPV+ are referred for colposcopy. Women who have discordant results receive repeat testing with an HPV test and are referred to colposcopy if they are HPV+. Women who have an initial or subsequent normal test result are assumed to return to routine screening.

^5 ^Women who are HPV+ are assumed to have a Pap test. Women with an ≥ ASC-US Pap result are referred for colposcopy. Women with discordant test results are assumed to receive another HPV test and are referred to colposcopy if they are HPV+. Women with an initial or subsequent normal test result are assumed to return to routine screening.

**Table 2:** Selected estimates for screening test accuracy, costs and utilities.

Screening Adherence ^(1)^			
<20	0.5-0.75 (0.35-1.0)		
20-29	0.5-0.8 (0.35-1.0)		
30-39	0.7-0.9 (0.5-1.0)		
40-49	0.6-0.9 (0.4-1.0)		
50+	0.3-1.0 (0.2-1.0)		
Screening Test Accuracy [8,21]^1,2^			
	Age <30	Age 30+	
Pap sensitivity for CIN 1+	0.42 (0.31-0.72)	0.32 (0.30-0.66)	
Pap specificity for <CIN 1	0.98 (0.80-0.95)	0.94 (0.82-0.95)	
HPV sensitivity for CIN 1+	0.83 (0.68-1.0)	0.71 (0.65-1.0)	
HPV specificity for <CIN 1	0.83 (0.80-0.97)	0.97 (0.94-1.0)	
HPV and Pap sensitivity for CIN 1+	0.88 (0.72-1.0)	0.75 (0.69-1.0)	
HPV and Pap specificity for <CIN 1	0.82 (0.79-0.96)	0.95 (0.92-1.0)	
Costs in Canadian $ (2006)[26-29,31]^3^			
	Canada and Ontario	Alberta	Newfoundland
Conventional Pap	$28 ($14-$56)	$30 ($15-$59)	$29 ($14-$58)
Liquid-based Pap	$32 ($16-64)	$35($17-$70)	$33 ($16-$65)
HPV test (hc2)	$53 ($14-$106)	$50 ($14-$100)	$53 ($14-$106)
Colposcopy + biopsy	$337 ($168-$673)	$376 ($188-$752)	$412 ($206-$824)
LEEP	$965 ($83-$1930)	$1082 ($541-$2164)	$1044 ($522-$2088)
Stage I Cancer	$11153 ($5576-$22305)	$12126 ($6063-$24253)	$11898 ($5949-$23797)
Stage II - III Cancer	$17644 ($8822-$35288)	$19185 ($9592-$38369)	$18824 ($9412-$37648)
Stage IV - Cancer	$24110 ($12055-$48220)	$26215 ($13107-$52430)	$25722 ($21861-$51445)
Utilities[32]^4^			
False-positive screening test result	-.02		
Duration	2 1/2 months[8]		
Stage I Cancer	0.76		
Duration	5 years ^5^		
Stage II - IV Cancer	0.67		
Duration	5 years ^5^		

^1 ^Ranges used for beta distributions for probabilistic sensitivity analyses

^2 ^Estimates of sensitivity assumed to be the same for LBC; specificity estimated to be 0.84[23] of base estimate of specificity for conventional Pap.

^3 ^Ranges used for normal distributions for costs for probabilistic sensitivity analyses

^4 ^Cost per quality-adjusted life-year calculated in sensitivity analyses

^5 ^Women who are alive at the end of 5 years are assumed to enter the cancer survivor state. The utility for a cancer survivor is assumed to be 1.

#### Screening test(s) sensitivity and specificity

Estimates of sensitivity and specificity were from a recently published randomized trial comparing HPV and conventional Pap tests [8,9]. Since the trial enrolled women aged 30+, we used estimates from another study that corrected for verification bias and provided estimates of test accuracy stratified by age <30 or 30+ to determine the sensitivity and specificity in women aged <30 years [21] Given a move towards considering CIN 2-3 as the disease state that is more likely to be a cancer precursor, and CIN 1 as a marker of HPV infection, we examined two different thresholds for disease: CIN 1+ and CIN 2+ [22]. Conventional Pap smears were assumed for the base case analysis; in sensitivity analyses we assumed that liquid-based Paps would have approximately the same sensitivity and lower specificity compared to conventional Paps, based on the results of a randomized controlled trial [23]. We did not model the impact of either type of Pap smear on inadequate results. To address concerns that the estimates of sensitivity for CIN 2+ for both Pap and HPV reported in CCCaST may be lower than those based on actual practice patterns in Canada, in sensitivity analyses, we examined the impact of using more commonly accepted estimates of sensitivity of Pap and HPV tests for detection of CIN 2+: 55.6% and 95% respectively [24,25].

#### Costs

The costs (Table 2) for Pap and HPV tests including laboratory costs were obtained from provincial fee schedules and communications with selected laboratories [26-28]. Professional costs for diagnostic procedures were also obtained from provincial fee schedules, while technical costs were based on data from the Ontario Case Costing Initiative website [26-29]. These were then adjusted for Alberta and Newfoundland by using provincial costs per weighted case (CPWC) as reported by the Canadian Institute of Health Information (CIHI) [30]. Costs for cancer care by stage were based on figures from Brisson et al. (2007), and were also adjusted for each province using CIHI's CPWCs [31]. Costs from Ontario were assumed to reflect those for the country as a whole. All estimates were measured in 2006 Canadian dollars. The triage test strategies were assumed to be conducted at two separate visits. Both tests were assumed to be collected at a single visit for the combined test procedure.

#### Utilities

For the model, previously published utility estimates for a false positive Pap screening test result and cancer (Stage I, Stage II-IV) were used (Table 2); these estimates were assumed to be constant by age [32]. The false-positive test result was assumed to be applicable to both Pap and HPV tests. Since these estimates were derived from a small study of college-aged women in the U.S., we examined the impact of quality-adjusted life years on cost-effectiveness results in sensitivity analyses only.

#### Analysis

The analysis was conducted from a health-system perspective. We calculated incremental cost-effectiveness ratios (ICERs) in which the average lifetime costs and life-expectancy or quality-adjusted life-expectancy of a strategy were compared with an adjacent strategy. Strategies that were more costly and less effective or less costly and less cost-effective than an adjacent strategy were considered to be dominated. We adjusted future costs and life expectancy to current values by discounting them at 3% annually. In sensitivity analyses, we calculated cost per quality-adjusted life year. In addition to one-way sensitivity analyses, in which key parameters, including test accuracy estimates, adherence to screening and costs were varied, a probabilistic sensitivity analysis was also performed on strategies consistently identified as cost-effective to explore the impact of the joint uncertainty surrounding model parameters. This analysis was undertaken by assigning probability distributions to key model parameters (costs and estimates of sensitivity and specificity) and subsequently propagating this uncertainty through the model using Monte Carlo sampling techniques to produce information on the likelihood that each intervention produces the greatest amount of net benefit at different willingness to pay thresholds. The results of these simulations are presented as cost-effectiveness acceptability curves (CEACs).

## Results

### Model calibration

**Figure 1:**
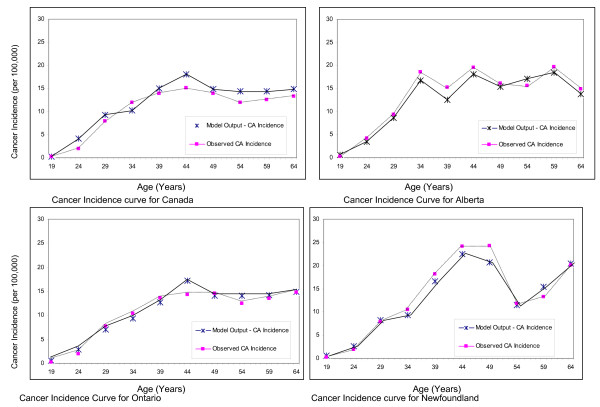
**Model estimated and observed age-specific cancer incidence curves for Canada and three provinces: Alberta, Ontario and Newfoundland**.

Figure (1a - d) presents the model predicted and observed age-specific cancer incidence rates. All provinces show a similar pattern of a steadily increasing incidence, with a peak in the mid-40s and a leveling off or fluctuations in incidence occurring after that. Of the three provinces, Newfoundland has the highest observed cancer incidence.

### Base case results

Table 3 presents the expected lifetime number of false positives and cancers per 100,000 women associated with the different screening strategies (for Ontario; results were similar for the other provinces and Canada as a whole). Strategies identified as cost-effective are presented in bold. As shown, the Miller strategy was associated with the highest expected number of cancers. Strategies of HPV testing followed by Pap triage were associated with fewer false-positive results and fewer cancers compared to strategies that used the Miller recommendations prior to age 25, and then switched to another strategy, or HPV-only strategies (conducted at less frequent screening intervals). Of note, strategies conducted beginning at age 18 were associated with a much higher number of false-positives, but slightly fewer cancers compared to the same strategies starting at age 25.

**Table 3:** Model predicted cancer (all stages) and false positive test results per 100,000 (Ontario).

Strategy	False Positives	Cancer (All Stages)
**No Intervention (Natural history) **^1^	**-**	**2,145**
**HPV with Pap Triage, q5, age 25^1^(SS 26)**	**2,871**	**736**
**HPV with Pap Triage, q3, age 25^1^(SS 23)**	**5,585**	**467**
HPV with Pap Triage, q5, age 18 (SS 25)	7,044	679
**HPV with Pap Triage, q3, age 18^1 ^(SS 22)**	**9,872**	**437**
Miller at age 18, then HPV with Pap Triage, q5, age 25 **(SS 27)**	11,404	658
Miller at age 18, then HPV with Pap Triage, q3, age 25 **(SS 24)**	14,160	411
Miller strategy (Pap q1 beginning at age 18, then q3 at age 21) **(SS 1)**	20,529	809
HPV only, q5, age 25 **(SS 8)**	22,437	586
Pap only, q2, age 18 **(SS 3)**	25,103	600
Pap with HPV Triage, q1, age 25 **(SS 20)**	27,660	364
Pap and HPV, q5, age 25 **(SS 17)**	30,817	548
**Miller at age 18, then Pap with HPV Triage, q1, age 25^1^(SS 21)**	**37,034**	**330**
HPV only, q3, age 25 **(SS 5)**	40,789	361
HPV only, q5, age 18 **(SS 7)**	40,957	538
**Pap with HPV Triage, q1, age 18^1^(SS 19)**	**44,903**	**309**
**Pap only, q1, age 18^1^(SS 2)**	**46,337**	**303**
Pap and HPV, q5, age 18 **(SS16)**	50,790	500
Pap and HPV, q3, age 25 **(SS 14)**	54,835	335
HPV only, q3, age 18 **(SS 4)**	60,456	330
Miller at age 18, then HPV only, q5, age 25 **(SS 9)**	61,375	517
Miller at age 18, then Pap and HPV, q5, age 25 **(SS 18)**	73,470	481
Pap and HPV, q3, age 18 **(SS 13)**	76,438	304
Miller at age 18, then HPV only, q3, age 25 **(SS 6)**	79,887	301
Pap and HPV, q2, age 25 **(SS 11)**	82,340	229
Miller at age 18, then Pap and HPV, q3, age 25 **(SS 15)**	97,645	283
**Pap and HPV, q2, age 18^1^(SS 10)**	**118,646**	**180**
Miller at age 18, then Pap and HPV, q2, age 25^2 ^**(SS 12)**	125,229	184

^1 ^Strategy falls on the efficiency frontier (for Ontario)

**Figure 2:**
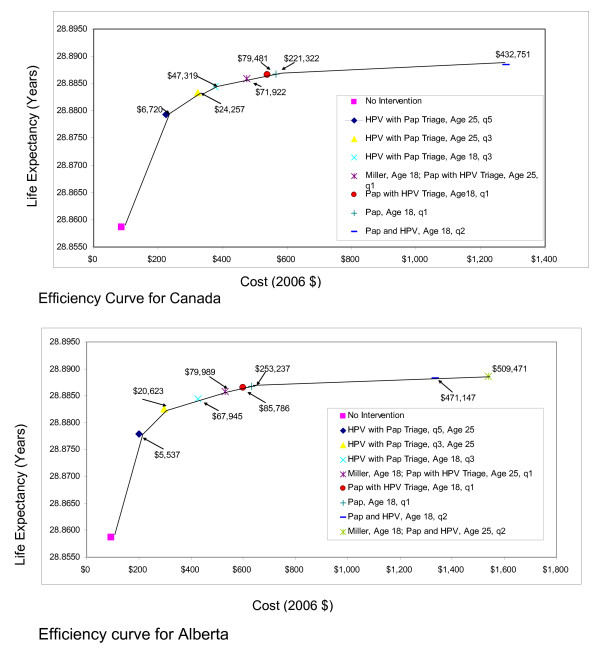
**Efficiency curves for Canada and the province of Alberta**.

**Figure 3:**
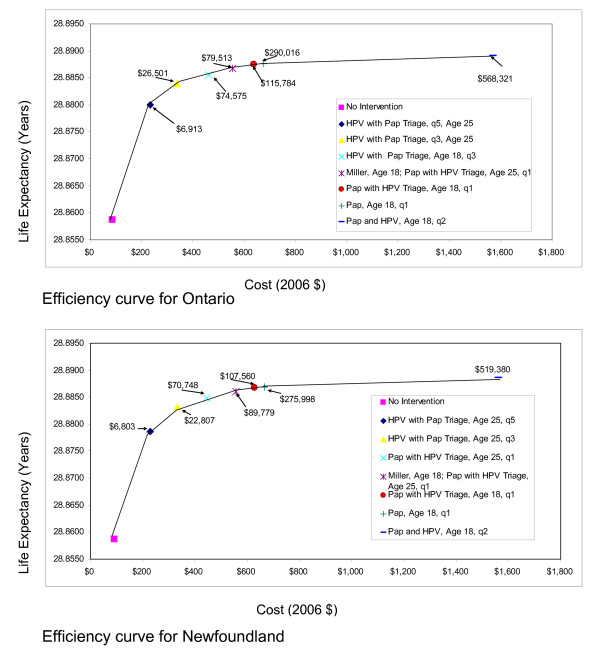
**Efficiency curves for the provinces of Ontario and Newfoundland**.

Figures 2 and 3 present the efficiency curves for the three different Provincial models and the Canada model. The strategies identified as cost-effective were similar for the four models. HPV with Pap triage conducted every three or five years was associated with ICERs <$50,000 per life year gained. Pap followed by HPV triage was also associated with ICERs that were <$100,000 per life year gained. Of note, the currently recommended strategy of screening yearly beginning at age 18 and then every three years from age 21 (Miller strategy) was consistently more costly and less effective (dominated) than a strategy of HPV with Pap triage, beginning at age 25 and conducted every three years.

### Sensitivity analyses

Results were similar to those obtained for the base case when adherence to screening was varied, liquid-based cytology was assumed instead of conventional Paps, HPV triage was assumed for women who had ASC-US Pap results instead of repeat Pap smears, costs for HPV DNA testing, colposcopy and biopsy or treatment for CIN or cancer were varied over a wide range or the outcome was cost per quality-adjusted life year instead of cost per life year. Results were also similar to those obtained for the base case for a range of estimates of sensitivity and specificity including the use of more commonly observed estimates of sensitivity for conventional Paps and HPV for detection of CIN 2+. The results were sensitive to the choice of discount rate, with HPV with Pap triage, beginning at age 25 conducted every five years dominating the Miller strategy when the discount rate was low (approximately <2.0%); at higher rates, the same strategy was identified as cost-effective compared to the Miller strategy, although it should be noted that the differences in life-expectancy were small (<1/2 day). If the cost of the Pap was reduced by half to approximately $14, the Miller strategy was the cheapest option; screening strategies using Pap with HPV triage conducted every year beginning at age 25 or age 18 were associated with ICERs of less than $100,000 per LY.

**Figure 4:**
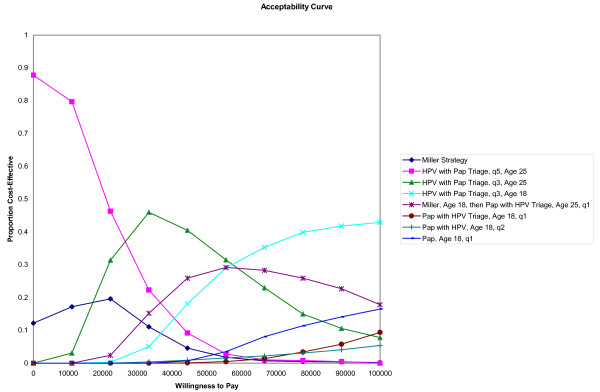
**Acceptability curves for strategies identified as cost effective (for Ontario - results for other provinces and Canada are similar)**.

The results of the probabilistic sensitivity analysis are presented in Figure 4 (for Ontario) in the form of cost-effectiveness acceptability curves. The frontier is formed by selecting only those strategies that have the greatest net benefits per willingness to pay threshold. As shown, if a decision maker is willing to pay between $20,000 and $50,000 per LY, a strategy of HPV testing followed by Pap triage for HPV positive women, conducted every three years, beginning at age 25 would provide the greatest net benefit. If willingness to pay is below $20,000 per LY, the same strategy of HPV with Pap triage conducted every five years is identified as providing the greatest net benefit.

## Discussion

Our findings suggest that a strategy of screening every three years, beginning at age 25, with HPV testing first followed by Pap triage for women with positive HPV results, and referral to colposcopy for any woman with an ≥ ASC-US Pap result may be more effective at preventing cancer, and less costly than the currently recommended strategy of screening every year beginning at age 18 and then every three years at age 21 with referral to colposcopy for any woman with an ≥ low grade squamous intra-epithelial lesion Pap result, and repeat testing for women with ASC-US Pap results. These findings were consistent at both the Provincial level and for Canada as a whole.

These findings reflect those of Mayrand et al. 2007, who showed that a strategy of HPV testing followed by Pap triage would be more sensitive and as specific as a strategy of sending everyone to colposcopy with a test result of ≥ LSIL, but less sensitive and more specific than a strategy of sending everyone with an ≥ ASC-US test result to colposcopy [8,9]. This is because the currently recommended strategy for screening in Canada is a hybrid: women with ASC-US Pap test results have repeat exams instead of being sent immediately to colposcopy; women with ≥ LSIL are referred for immediate colposcopy. The strategy of HPV testing followed by Pap triage was more effective because all women with a positive test result for both were sent to colposcopy, and women with a negative HPV test result were assumed to return to routine screening. We assumed that women with discordant results would receive a repeat HPV test and be referred for colposcopy if positive, or return to routine screening if negative. This strategy thus avoids the increased costs of screening women at an early age, but increases the likelihood of detecting significant disease by using HPV testing first, followed by Pap testing. Our findings are similar to those of Goldhaber-Fiebert et al. (2008) who showed that in the US, triennial Pap-based screening with HPV triage, beginning at age 21, with a switch to HPV testing with Pap triage at age 30 years was cost-effective [33].

There are limitations to this analysis. The first is the lack of data from a single Canadian study that provides estimates of HPV and Pap screening test performance by age (<30, 30+). To address this we used data from other studies that corrected for verification bias [3,21]. Our findings were robust across a range of estimates for sensitivity and specificity. Another potential limitation of this analysis is that the differences in cancer incidence between the provinces may be due to other differences besides adherence; however to our knowledge, there are no published studies that have explored the basis for provincial differences [2]. Finally, we did not include vaccination in our analysis. The analysis by Goldhaber-Feibert et al. also examined screening in combination with HPV vaccination; they concluded that for a cohort of vaccinated girls, a strategy of Pap-based screening with HPV triage beginning at age 25 with a switch to HPV with Pap triage at age 35 conducted either every three or every five years may be cost-effective [33]. However, given the lack of long-term data on the performance of Pap and HPV-based cervical cancer screening in the era of vaccination, and different decisions at the Provincial level regarding the schedule for dosing for HPV vaccination, it is currently unclear what changes will be needed, if any [34]. In terms of expected technical performance, strategies based on HPV testing with Pap triage may be more likely to suit cervical cancer screening needs post-vaccination [35].

## Conclusion

In conclusion, our results suggest that a new approach to screening that combines HPV testing with triage using Pap smears, conducted every three years, beginning at age 25 may be associated with fewer cancers and lower costs than the currently recommended strategy of screening every year from age 18 to age 21, and then every three years with cytology only.

## Abbreviations

ASC-US: atypical squamous cells of undetermined significance; CCCaST: Canadian Cervical Cancer Screening Trial; CEACs: Cost-Effectiveness Acceptability Curves; CIN: Cervical Intraepithelial Neoplasia; CPWC: Costs per Weighted Case; CIHI: Canadian Institute of Health Information; HPV: human papillomavirus; LSIL: low grade intraepithelial neoplasia; ICER: incremental cost-effectiveness ratios.

## Competing interests

Shalini Kulasingam has previously received research support from Merck, Inc and CSL-Australia. She has also served as a consultant for CSL-New Zealand and Sanofi-Pasteur MSD. Shalini Kulasingam is currently a consultant for Medtronic. Raghu Rajan, Yvan St. Pierre and C. Victoria Atwood have no competing interests. Evan Myers has received research funding and served as a consultant for Merck, Inc, and as a consultant for GlaxoSmithKline. Eduardo Franco has served in advisory boards or as a consultant for companies involved with HPV vaccination (GlaxoSmithKline and Merck), HPV diagnostics (Roche, Qiagen, Gen-Probe), and cervical cancer screening (Cytyc, Ikonisys). He has also received unconditional research grant support from Merck for an investigator-initiated molecular epidemiology project.

## Authors' contributions

SLK participated in the design of the study, developed the cost-effectiveness models, conducted the cost-effectiveness analysis and drafted the manuscript. RR conceived of the study, participated in the design of the study, the acquisition of data for use in the cost-effectiveness models and critically reviewed the manuscript for important intellectual content. YSP participated in the design of the study, the acquisition of data for use in the cost-effectiveness models and critically reviewed the manuscript for important intellectual content. CVA participated in the design of the study, the acquisition of data for use in the cost-effectiveness models and critically reviewed the manuscript for important intellectual content. ERM participated in the design of the study, the development of the cost-effectiveness models and critically reviewed the manuscript for important intellectual content. ELF conceived of the study, participated in the design of the study, the acquisition of data for use in the cost-effectiveness models and critically reviewed the manuscript for important intellectual content. All authors have read and approved the final manuscript.

## Pre-publication history

The pre-publication history for this paper can be accessed here:

http://www.biomedcentral.com/1741-7015/7/69/prepub
